# Unsuppressed Viremia and Lower CD4 Count Associated With Faster Telomere Attrition in African Children With Perinatal Human Immunodeficiency Virus on Long-term Antiretroviral Therapy

**DOI:** 10.1093/infdis/jiag060

**Published:** 2026-02-07

**Authors:** Julian P G Shellard, Emily Carr, Gianni Tam-McMillan, Emma W Mao, Hilda Mujuru, Hildah Banda Mabuda, Molly Chisenga, Tsitsi Bandason, Nyasha V Dzavakwa, Lackson Kasonka, Victoria Simms, Celia L Gregson, Rashida A Ferrand, Sarah L Rowland-Jones, Anthony Y Y Hsieh

**Affiliations:** Centre for Immuno-Oncology, Nuffield Department of Medicine, ⁠University of Oxford, Oxford, United Kingdom; Institute for Infection and Immunity, City St George's, University of London, London, United Kingdom; Centre for Immuno-Oncology, Nuffield Department of Medicine, ⁠University of Oxford, Oxford, United Kingdom; Centre for Immuno-Oncology, Nuffield Department of Medicine, ⁠University of Oxford, Oxford, United Kingdom; Department of Paediatrics, University of Zimbabwe, Harare, Zimbabwe; University Teaching Hospital, Women and Newborn Hospital, University of Zambia, Lusaka, Zambia; University Teaching Hospital, Women and Newborn Hospital, University of Zambia, Lusaka, Zambia; Health Research Unit Zimbabwe, Biomedical Research and Training Institute, Harare, Zimbabwe; Health Research Unit Zimbabwe, Biomedical Research and Training Institute, Harare, Zimbabwe; International Statistics and Epidemiology Group, Faculty of Epidemiology and Population Health, London School of Hygiene and Tropical Medicine, London, United Kingdom; University Teaching Hospital, Women and Newborn Hospital, University of Zambia, Lusaka, Zambia; Health Research Unit Zimbabwe, Biomedical Research and Training Institute, Harare, Zimbabwe; International Statistics and Epidemiology Group, Faculty of Epidemiology and Population Health, London School of Hygiene and Tropical Medicine, London, United Kingdom; Health Research Unit Zimbabwe, Biomedical Research and Training Institute, Harare, Zimbabwe; Global Musculoskeletal Research Group, Musculoskeletal Research Unit, Bristol Medical School, University of Bristol, Bristol, United Kingdom; Health Research Unit Zimbabwe, Biomedical Research and Training Institute, Harare, Zimbabwe; Clinical Research Department, Faculty of Infectious and Tropical Diseases, London School of Hygiene and Tropical Medicine, London, United Kingdom; Centre for Immuno-Oncology, Nuffield Department of Medicine, ⁠University of Oxford, Oxford, United Kingdom; Centre for Immuno-Oncology, Nuffield Department of Medicine, ⁠University of Oxford, Oxford, United Kingdom; Chinese Academy of Medical Sciences Oxford Institute, Nuffield Department of Medicine, University of Oxford, Oxford, United Kingdom

**Keywords:** HIV/AIDS, pediatrics, aging, telomere

## Abstract

**Background:**

Human immunodeficiency virus (HIV-1) infection leads to reduced telomere length (TL), a biomarker of immune aging. We investigated relationships between HIV viral load (VL) and CD4 count with TL and its rate of attrition in children with HIV from Zambia and Zimbabwe.

**Methods:**

Buffy coat was obtained at baseline and 48 weeks from children aged 11–19 years with perinatally acquired HIV taking combination antiretroviral therapy (cART) for >6 months recruited into the VITALITY trial. Relative TL was measured using monochrome multiplex quantitative polymerase chain reaction, standardizing units for analysis. Cross-sectional analyses used multivariable linear regression adjusting for age, sex, and study site; longitudinal analysis additionally adjusted for baseline TL.

**Results:**

Among participants at baseline (N = 842; mean ± SD age, 15.5 ± 2.6 years; 53.2% female), 678 (80.5%) had HIV VL <60 copies/mL, 66 (7.8%) had 60–1000 copies/mL, and 98 (11.6%) had >1000 copies/mL. The mean CD4 count was 584 ± 243 cells/μL. Compared to participants with VL <60 copies/mL, those with VL >1000 copies/mL had shorter TL (β = −.239 [95% confidence interval {CI}, −.451 to −.026]; *P* = .028), whereas those with 60–1000 copies/mL did not (*P* = .836). Lower CD4 cell count was associated with shorter TL (β = −.038 [95% CI, −.009 to −.066] per 100 CD4 cells/μL; *P* = .009). In longitudinal analysis (n = 783) after mean 336 ± 6 days, those with HIV VL >1000 copies/mL at both timepoints had an accelerated telomere attrition rate (β = −.276 [95% CI, −.546 to −.005]; *P* = .046) compared with participants with VL <1000 copies/mL. Lower baseline CD4 count was associated with faster telomere attrition rate (β = −.033 [95% CI, −.008 to −.057]; *P* = .009).

**Conclusions:**

HIV VL >1000 copies/mL among children with HIV on cART in Africa is associated with a degradation of immune age within 1 year, which may increase risk of comorbidities later in life.

**Clinical Trials Registration:**

PACTR202009897660297.

## Teaser

Measurable immunologic aging longitudinally within 1 year and prevalence of unsuppressed viremia despite long-term antiretroviral therapy highlight the vulnerability of African children with HIV to poorer health longevity. Timely strategies to establish viral control and promote healthy aging are required.

Over 90% of the 1.7 million children with perinatally acquired human immunodeficiency virus (HIV-1) live in Africa [1]. The global scale-up of combination antiretroviral therapy (cART) has reduced progression to AIDS in children with HIV (CWH), and therefore growing numbers are reaching adulthood. However, despite cART, CWH may not experience healthy aging, with an increased risk of developing long-term comorbidities such as chronic lung disease [2], cardiovascular abnormalities [3], and impaired growth [4]. Immunologic deterioration may explain the development of comorbidities in people living with HIV [5], and CWH could be disproportionately affected given that HIV is acquired before maturation of the immune system is complete [6].

Both adults and children with HIV on cART exhibit premature age-associated immunologic deterioration [5, 7]. While precise mechanisms are unknown, HIV infection is associated with suppressed thymic output [8] and an accumulation of senescent CD8 T-cell [9] and B-cell [10] populations. Disruption to the homeostasis between regulatory and activated T-cell subsets [11] and coinfections with chronic viruses such as cytomegalovirus (CMV) [12] contribute to persistent inflammation that may underlie immune dysregulation. Compared to adults in resource-rich settings, CWH in Africa remain an understudied population despite their rising burden of non-AIDS age-associated complications. Immune aging studies accounting for ethnicity, nutrition, chronic infections, and socioeconomic status (SES) are needed across different global populations.

There is robust evidence that accelerated immune aging in children and adults with HIV is reflected in shorter leukocyte telomere length (TL), which is exacerbated among those with high viral load (VL) [9, 13] and low CD4 cell count [14]. Protecting the ends of chromosomes against nonhomologous end-joining, telomeres are nucleoprotein complexes that shorten with each cell replication [15]. Various factors modulate telomere shortening, ranging from sex, genetics, and ethnicity to environment and other determinants of health. TL is predictive of mortality [16] and poorer health outcomes, particularly cardiovascular disease [17], later in life. It is plausible that vitamin D concentrations associate with TL [18], and data from a randomized controlled trial suggest that vitamin D_3_ supplementation decreases telomere attrition rate in adults [19]. Additionally, the rate of telomere attrition is associated with cardiovascular damage [20] and mortality [21] in later life and predictive of poorer midlife cognition [22]. There are limited studies investigating the link between HIV control and TL in CWH from Africa, and none have measured longitudinal TL attrition rate.

This study aimed to assess whether TL was associated cross-sectionally with HIV VL and CD4 T-cell count in CWH from Zambia and Zimbabwe. Longitudinally, we assessed whether the telomere attrition rate over 48 weeks was associated with the HIV VL trajectory across both visits and baseline CD4 count.

## MATERIALS AND METHODS

### Study Population

**Figure 1.**
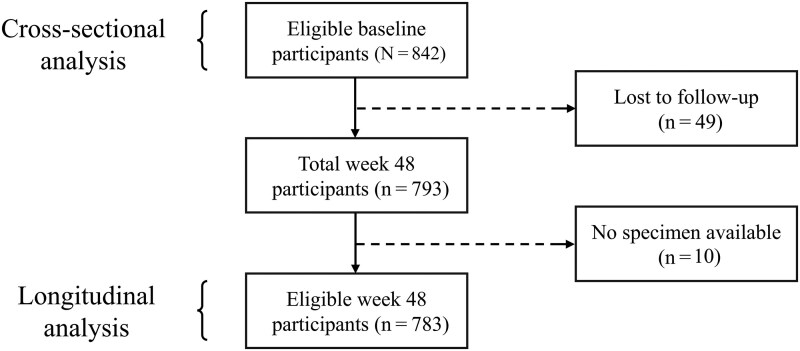
Flowchart of VITALITY study participants.

This study is nested within a phase 3, 2-site, individually randomized, double-blinded, placebo-controlled trial of vitamin D_3_/calcium carbonate supplementation (Vitamin D_3_ and calcium carbonate supplementation for adolescents with HIV to reduce musculoskeletal morbidity and immunopathology [VITALITY]; clinical trials registration: PACTR202009897660297), described previously [23]. Participants were 11–19 years of age with perinatally acquired HIV, taking cART for >6 months, and recruited from the University Teaching Hospital (Lusaka, Zambia) and Sally Mugabe Central Hospital (Harare, Zimbabwe). Recruitment occurred between February and November 2021. All participants had baseline blood specimens, but those with no specimen at 48 weeks or loss to follow-up were excluded from longitudinal analysis (Figure 1).

### Ethical Approval

Ethical approval was granted by the Biomedical Research and Training Institute Institutional Review Board (AP158/2020), the Medical Research Council of Zimbabwe (A/2626), the University of Zambia Biomedical Research Ethics Committee (1116–2020), and the London School of Hygiene and Tropical Medicine Ethics Committee (22030).

### Data Collection

cART history and SES were collected by questionnaire as described previously [24]. 25-hydroxyvitamin D (25(OH)D) was measured by liquid chromatography–tandem mass spectrometry and HIV VL by polymerase chain reaction (PCR), as described previously [24]. CD4 count was measured using an Abbott CD4 PIMA analyzer. CMV immunoglobulin G (IgG) was measured for Zimbabwean participants using the Anti-CMV IgG Human ELISA Kit (catalog number Ab108724, Abcam, Cambridge, UK) following the manufacturer's instructions.

### Telomere Length Measurement

Genomic DNA was purified from 200 μL of buffy coat cells and eluted in 60 μL using the “spin protocol” for the QIAamp DNA Blood Mini Kit (catalog number 51106, Qiagen, Hilden, Germany), following the manufacturer's recommendations.

Relative TL of extracted DNA was measured by monochrome multiplex quantitative PCR (MMqPCR) using the QuantStudio 1 Real-Time PCR System (Thermo Fisher, Waltham, Massachusetts, USA) and performed with quality control as previously described [25]. MMqPCR measures the quantities of telomeric repeat sequences (T) and of albumin, a single nuclear gene (S). The calculated T/S ratio is a relative measurement proportionate to TL in each cell.

The highest point of the standard curve (range, 0.16–20 ng/μL) was created using DNA pooled from 3 volunteer donors. Extracted VITALITY DNA (range, 5.70–2026.58 ng/μL) was diluted within the standard curve range. Two internal controls and a negative control were run on each plate. Specimens were run in duplicate. Internal controls were generated with DNA extracted from 10 pooled VITALITY specimens (5 ng/μL) and HEK293T cells (1 ng/μL). Fluorescence data were exported using Design and Analysis Software 2.6.0 (Thermo Fisher) and cycle thresholds were generated using LinRegPCR as previously described [25].

Baseline specimens were assayed in a randomized order, after which all available follow-up specimens underwent the same process. This totaled 46 MMqPCR runs and the interassay coefficient of variation for the 2 internal controls was 10% and 7%.

### Statistical Analyses

TL and telomere attrition rate, calculated as the difference in TL between clinic visits divided by the time between visits, are based on relative measurements. Therefore, both were standardized such that effect sizes represent standard deviations (SD) within the sample. At baseline, cross-sectional associations between HIV VL and CD4 count, and TL were examined. In longitudinal analysis, associations were assessed between telomere attrition rate and HIV VL trajectories across both visits, as well as baseline CD4 count. When constructing multivariable models, factors known to confound the relationship between TL and HIV were selected a priori and considered for adjustment. Age and sex were always adjusted for, given their clear relationship with TL. Then, body mass index (BMI), age at cART initiation, cART regimen (third drug class comprising protease inhibitors, nucleoside reverse transcriptase inhibitors, nonnucleoside reverse transcriptase inhibitors, or integrase strand transfer inhibitors [INSTIs]) [26], tenofovir disoproxil fumarate (TDF) usage [26], CMV IgG levels (only available in participants recruited in Zimbabwe) [27], SES quintile [28], 25(OH)D concentrations [18], and study site (Zambia vs Zimbabwe) were adjusted for if they were associated with TL in univariate analysis (*P* < .10). Using the final model, the effect sizes of VL groups were divided by the effect size of age to interpret the magnitude of observed differences in relative TL. The same variables were considered for longitudinal analysis, in addition to baseline TL. In previous literature, the telomere attrition rate has been shown to be overestimated due to the regression to the mean (RTM) effect when adjusting for baseline TL, so we undertook a RTM-corrected sensitivity analysis for longitudinal TL models as described previously [29]. In all models, collinearities and 2-way interactions were investigated and none were found. Statistical analyses were performed in GraphPad Prism software (version 10.1.2) and R (version 4.3.2, GUI 1.80 Big Sur ARM build).

HIV VL of <60 RNA copies/mL was defined as “undetectable,” and 1000 RNA copies/mL was the threshold for viral suppression in accordance with current World Health Organization guidance [30]. In cross-sectional analysis, VL was grouped into 3 categories: undetectable, 60–1000 RNA copies/mL, and >1000 RNA copies/mL (unsuppressed). In longitudinal analysis, 4 HIV VL “trajectories” are possible: suppressed to suppressed (S→S), suppressed to unsuppressed (S→U), unsuppressed to suppressed (U→S), and unsuppressed to unsuppressed (U→U). 25(OH)D concentrations were categorized as sufficient (≥75 nmol/L) or insufficient (<75 nmol/L), as described previously [24].

## RESULTS

### Study Participant Characteristics

A total of 842 and 783 blood biospecimens were available from participants at baseline (week 0) and follow-up (week 48), respectively (Figure 1). At baseline, 678 (80.5%) participants had undetectable viremia, 66 (7.8%) participants had 60–1000 RNA copies/mL, and 98 (11.6%) participants had >1000 RNA copies/mL (Table 1). Participants with VL >1000 RNA copies/mL were on average older and more often male than those with lower or undetectable VL. These participants were also less likely to be taking cART regimens that included INSTIs and TDF; however, age at cART initiation and cART duration did not differ between groups. 25(OH)D concentrations were higher among participants with 60–1000 RNA copies/mL, and this group had a slightly larger proportion of individuals in the highest SES quintile. BMI did not differ between VL groups.

**Table 1.** Study Participant Characteristics for the VITALITY Cohort

Characteristic	Undetectable (<60 RNA Copies/mL) (n = 678 [80.5%])	60–1000 RNA Copies/mL (n = 66 [7.8%])	>1000 RNA Copies/mL (n = 98 [11.6%])	*P* Value
Age, y	15 ± 3 (11–20)	16 ± 3 (11–20)	16 ± 2 (11–20)	<.001^a^
Female sex	367 (54)	43 (65)	38 (39)	.002^b^
BMI, kg/m^2^	18 ± 3 (12–30) (n = 676)	19 ± 3 (14–30)	19 ± 3 (12–27)	.900^a^
HIV VL, RNA copies/mL	NA	183 [90–399] (60–997)	18 380 [5499–61 445] (1015–7 848 680)	**<**.001^c^
Current CD4 T-cell count/µL blood	618 ± 230 (7–1631) (n = 676)	560 ± 245 (51–1227) (n = 65)	367 ± 216 (14–948) (n = 97)	<.001^a^
Age at cART initiation, y	5 [2–9] (0–19)	4 [2–8] (0–15)	6 [3–9] (0–15)	.225^d^
Duration of cART, y	10 ± 4 (1–19)	10 ± 4 (1–18)	10 ± 4 (2–17)	.158^a^
cART regimen (third drug class)				<.001^b^
PI	52 (8)	16 (24)	34 (35)	
NRTI/NNRTI	50 (7)	6 (9)	18 (18)	
INSTI	576 (85)	44 (67)	46 (47)	
TDF				<.001^b^
TDF in ART regimen	581 (86)	46 (70)	57 (58)	
Date of baseline blood specimen collection	26 Aug 2021 [14 Jun 2021–11 Oct 2021]	9 Jul 2021 [31 Mar 2021–28 Sep 2021]	29 Jun 2021 [13 Apr 2021–22 Sep 2021]	<.001^a^
SES				.051^b^
I (lowest)	134 (20)	12 (18)	24 (24)	
II	137 (20)	7 (11)	23 (23)	
III	147 (22)	14 (21)	14 (14)	
IV	136 (20)	11 (17)	15 (15)	
V (highest)	124 (18)	22 (33)	22 (22)	
CMV IgG, IU/mL	31 [27–35] (19–49) (n = 332)	33 [29–36] (18–51) (n = 41)	33 [30–39] (22–49) (n = 59)	.005^d^
Vitamin D, nmol/L 25(OH)D	63 [54–73] (21–126)	69 [59–83] (35–145)	65 [56–80] (27–135)	.002^d^
≥75 nmol/L	143 (21)	26 (39)	29 (30)	.001^b^
<75 nmol/L	535 (79)	41 (61)	68 (70)	
Zambian study site	356 (53)	25 (38)	39 (40)	.008^b^

Data are presented as No. (%), median [IQR] (range), or mean ± SD (range). CMV IgG data were only available for Zimbabwean participants.

Abbreviations: 25(OH)D, blood 25-hydroxy vitamin D level; ART, antiretroviral therapy; BMI, body mass index; cART, combination antiretroviral therapy; CMV, cytomegalovirus; HIV, human immunodeficiency virus; IgG, immunoglobulin G; INSTI, integrase strand transfer inhibitor; NA, not applicable; NNRTI, nonnucleoside reverse transcriptase inhibitor; NRTI, nucleoside reverse transcriptase inhibitor; PI, protease inhibitor; SES, socioeconomic status; TDF, tenofovir disoproxil fumarate; VL, viral load.

^a^Analysis of variance.

^b^Chi-square test.

^c^Mann–Whitney test.

^d^Kruskal–Wallis test.

### Correlates of Baseline Relative Telomere Length

**Figure 2.**
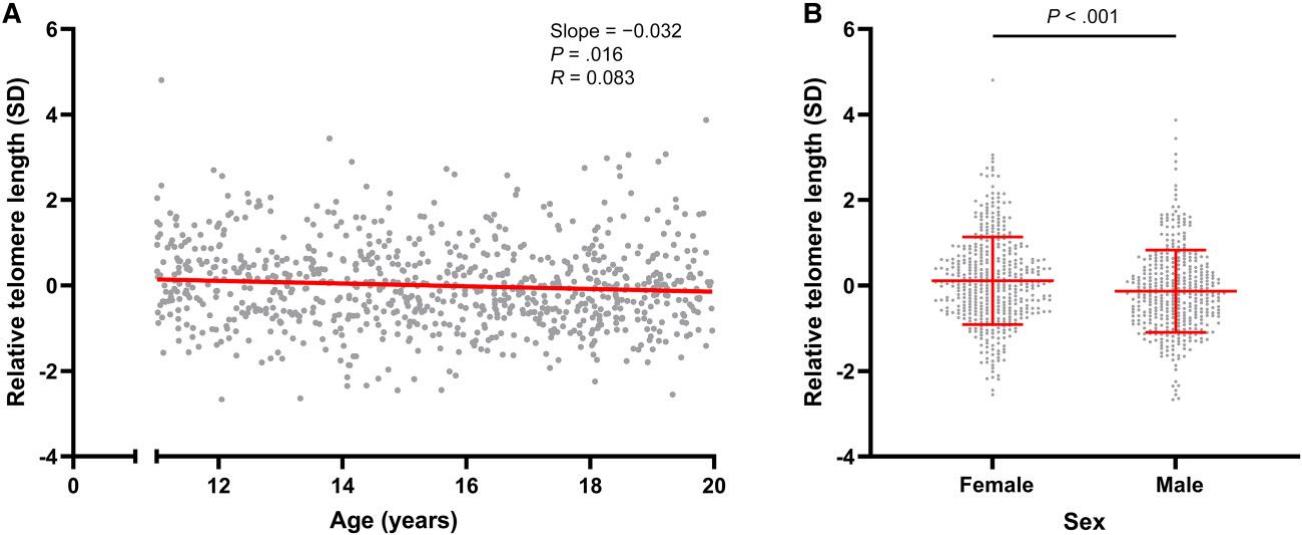
Baseline univariate associations of age and sex with relative telomere length (TL). *A*, Pearson correlation between age and TL (N = 842), line of best fit, slope, *P* value, and *R* value are shown. *B*, TL comparison between females and males (N = 842); mean and standard deviation (SD), unpaired *t*-test, and *P* value are shown.

TL declined with age (Figure 2*A*), with a negative slope of −0.032 SD/year (*P* = .016). On average, males had telomeres that were 0.247 SD shorter than females (*P* < .001) (Figure 2*B*). There was no evidence of association with BMI, SES quintile, cART regimen, TDF usage, and 25(OH)D in univariate TL analysis (Supplementary Table 1). Zambian participants had on average 0.218 SD longer TL than Zimbabwean participants (*P* = .002). There was no evidence of an association between CMV IgG and TL among Zimbabwean participants (slope = 0.011 SD/[IU/mL]) (*P* = .118).

**Figure 3.**
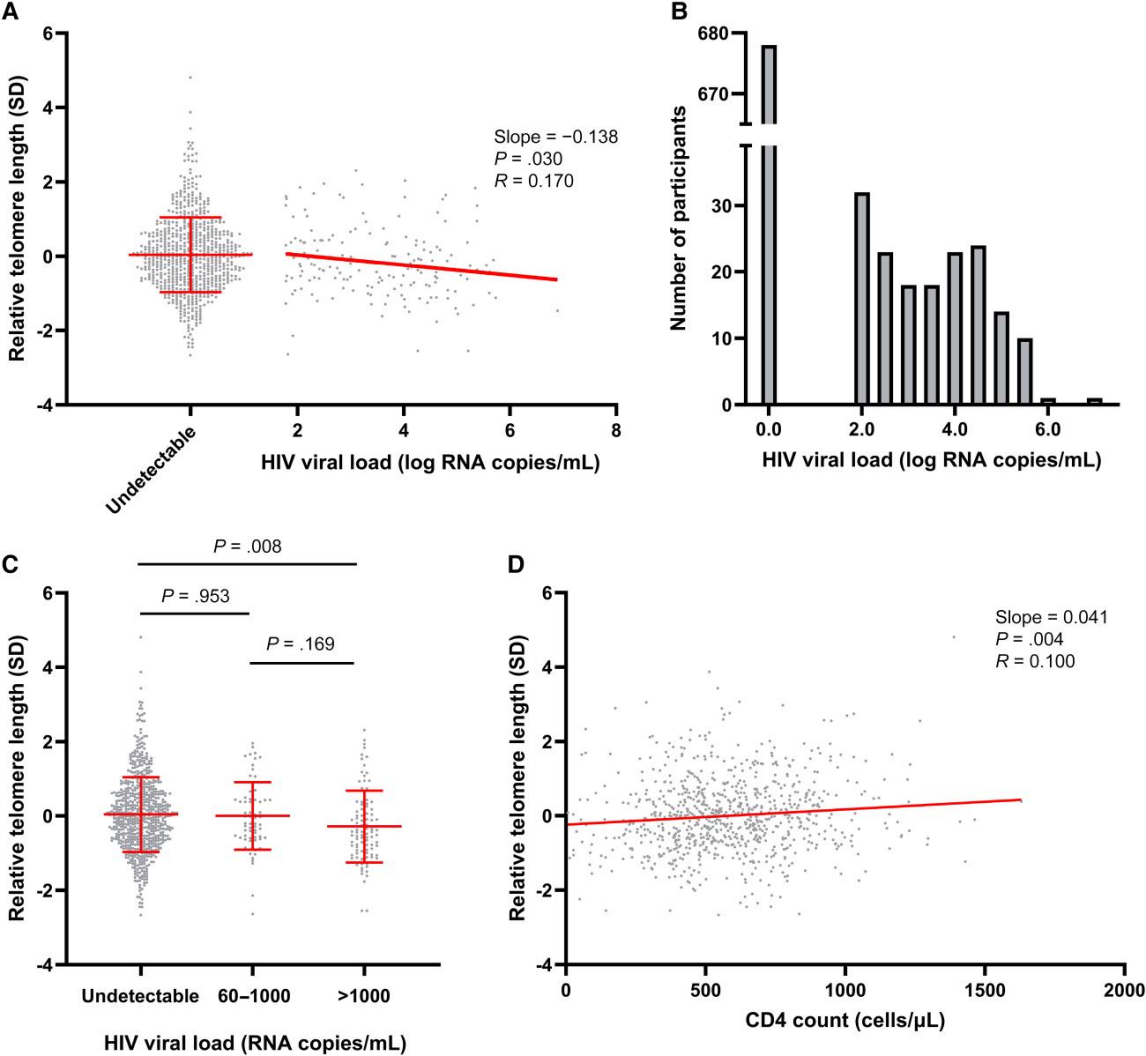
Baseline univariate associations of HIV viral load (VL) and CD4 count with relative telomere length (TL). *A*, Pearson correlation between log HIV VL and TL. Participants with an undetectable VL (<60 RNA copies/mL) are depicted as 1 category (n = 678); mean and standard deviation (SD) shown. Detectable participants are plotted on a continuous scale (n = 164); Pearson line of best fit, slope, *P* value, and *R* value are shown. *B*, Log HIV VL frequency distribution. Undetectable participants are plotted at 0 log RNA copies/mL. *C*, HIV VL against TL, wherein participants are categorized as undetectable (n = 678), VL 60–1000 RNA copies/mL (n = 66), or VL >1000 RNA copies/mL (n = 98). Mean and SD and Tukey posttest *P* values (analysis of variance adjusted for multiple comparisons) are shown. *D*, Pearson correlation between CD4 T-cell count and TL (n = 838); line of best fit, slope, *P* value, and *R* value are shown.

Participants with VL ≥60 RNA copies/mL had shorter TL than participants with undetectable VL at baseline (β = −.207 SD; *P* = .015). Among participants with VL ≥60 RNA copies/mL, TL decreased with increasing VL (slope = −0.138 SD/[log RNA copies/mL]) (*P* = .030; Figure 3*A*), and the VL frequency distribution of these participants (Figure 3*B*) had a trough approximating 3 log (1000) RNA copies/mL. Participants with >1000 RNA copies/mL had shorter TL than those with undetectable VL (Figure 3*C*). TL did not differ between participants with undetectable VL and those with 60–1000 RNA copies/mL. CD4 count and TL were positively associated (slope = 0.041 SD/[100 cells/μL]) (*P* = .004; Figure 3*D*).

We repeated this analysis in multivariable models and found that HIV VL >1000 RNA copies/mL was associated with shorter TL independent of age, sex, and study site (β = −.239 [95% confidence interval {CI}, −.451 to −.026]; *P* = .028) (Table 2). Based on the effect size between VL groups compared to the effect size of age, having VL >1000 RNA copies/mL was associated with shorter TL equivalent to approximately 11 years of aging, compared to undetectable VL. Adjusting for the same variables (age, sex, and study site), lower CD4 count was independently associated with shorter TL (β = −.038 [95% CI, −.009 to −.066] per 100 cells/μL; *P* = .009) (Table 2).

**Table 2.** Multivariable Linear Regression Models Investigating the Association of Telomere Length With HIV Viral Load or CD4 T-Cell Count at Baseline

HIV VL [Reference: Undetectable]	HIV VL Model				CD4 Count Model			
	Unadjusted		Adjusted^a^		Unadjusted		Adjusted^a^	
	β	*P* Value	β	*P* Value	β	*P* Value	β	*P* Value
>1000 copies/mL	−.321 (−.532 to −.109)	.003	−.239 (−.451 to −.026)	.028				
60–1000 copies/mL	−.0375 (−.290 to .215)	.770	−.026 (−.277 to .224)	.836				
CD4 count (per 100 cells/μL)					.041 (.013–.069)	.004	.038 (.009–.066)	.009

β and 95% confidence intervals are shown alongside *P* value. A negative β represents association with a shorter telomere length.

Abbreviations: HIV, human immunodeficiency virus; VL, viral load.

^a^Adjusted for age, sex, and study site.

### Correlates of Telomere Attrition Rate

Among the 783 participants with HIV VL measurements at both week 0 and 48, 648 (82.8%) participants were always suppressed (S→S) and 37 (4.7%) participants were always unsuppressed (U→U), with the threshold for suppression set at 1000 RNA copies/mL. Fifty (6.4%) participants were S→U and 48 (6.1%) participants were U→S (Supplementary Table 2).

**Figure 4.**
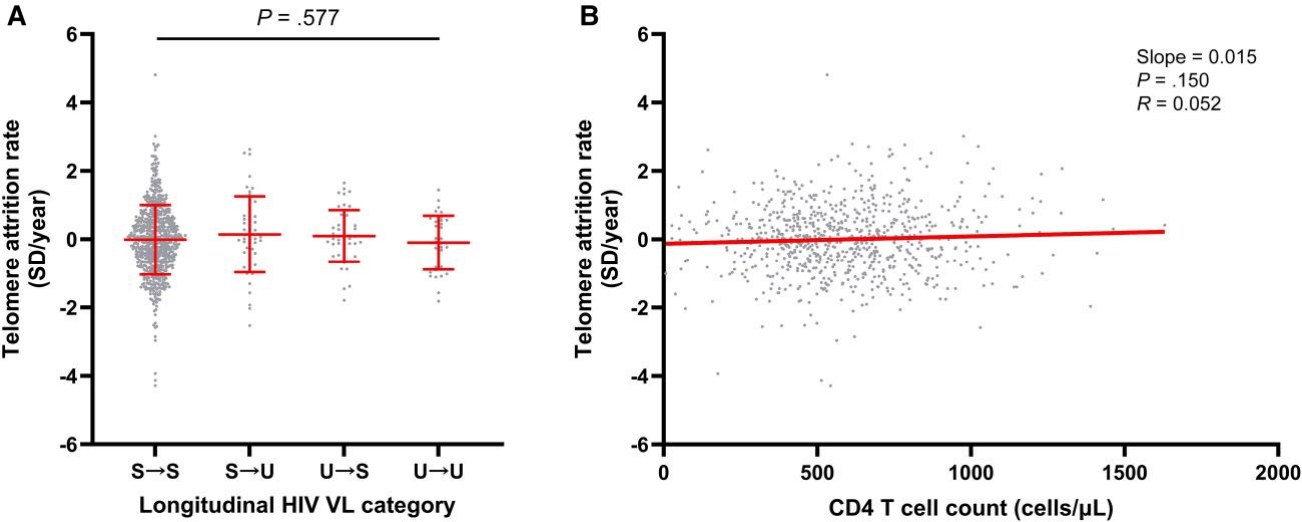
Longitudinal univariate analysis of telomere attrition rate. *A*, Telomere attrition rate against longitudinal HIV viral load groups, where participants are categorized as S→S (n = 648), S→U (n = 50), U→S (n = 48), or U→U (n = 37); S is classified as <1000 RNA copies/mL. Mean and SD and *P* value for analysis of variance are shown. (B) Pearson correlation between baseline CD4 T-cell count and telomere attrition rate (n = 780); Pearson line of best fit, slope, *P* value, and *R* value are shown. Abbreviations: S, suppressed; SD, standard deviation; U, unsuppressed; VL, viral load.

Telomere attrition rate was calculated as the difference in TL between weeks 0 and 48, divided by time between visits, then standardized. In univariate analysis, faster telomere attrition rate correlated with longer baseline TL (slope = −0.576, *P* < .001) and was faster in Zambian participants (β = −.345, *P* < .001) (Supplementary Table 3). Age, sex, and CMV IgG levels were not associated with telomere attrition rate (Supplementary Table 3). In unadjusted analyses, telomere attrition rates were similar across all longitudinal HIV VL trajectory groups (*P* = .577; Figure 4*A*). However, when adjusting for age, sex, baseline TL, and study site, telomere attrition rate was significantly increased in U→U participants compared with S→S participants (β = −.276 [95% CI, −.546 to −.005]; *P* = .046) (Table 3). Similarly, for CD4 T-cell count, univariate analysis showed no association with telomere attrition rate (Figure 4*B*), but after adjustment for the same variables (age, sex, and study site), a lower CD4 count at baseline correlated with faster telomere attrition rate (β = −.033 [95% CI, −.008 to −.057] per 100 cells/μL; *P* = .009) (Table 3). In the RTM sensitivity analysis, the accelerated telomere attrition rate for U→U participants was reproduced (Supplementary Table 4).

**Table 3.** Multivariable Linear Regression Models Investigating the Association of Telomere Attrition Rate With HIV Viral Load or CD4 T-Cell Count

HIV VL Trajectory [Reference: S→S]	HIV VL Model				CD4 Count Model			
	Unadjusted		Adjusted^a^		Unadjusted		Adjusted^a^	
	β	*P* Value	β	*P* Value	β	*P* Value	β	*P* Value
S→U	.158 (−.130 to .446)	.282	−.002 (−.235 to .232)	.989				
U→S	.113 (−.181 to .407)	.451	−.006 (−.245 to .233)	.960				
U→U	−.083 (−.415 to .249)	.624	−.276 (−.546 to −.005)	.046				
CD4 count (per 100 cells/μL)					.021 (−.008 to .051)	.624	.033 (.008–.057)	.009

β and 95% confidence intervals are shown alongside *P* value. A negative β represents association with a greater rate of telomere attrition.

Abbreviations: HIV, human immunodeficiency virus; S, suppressed; U, unsuppressed; VL, viral load.

^a^Adjusting for age, sex, baseline telomere length, and study site.

## DISCUSSION

To our knowledge, this is the first longitudinal study to demonstrate a greater rate of telomere attrition in children with HIV viremia >1000 RNA copies/mL despite long-term cART. In cross-sectional analyses, children with VL >1000 RNA copies/mL had a shorter baseline TL than children with undetectable VL. The magnitude of this TL difference was comparable to the magnitude of 11 years of average age-related TL decline in our models. Participants with sustained failure to control viral replication, defined by VL >1000 RNA copies/mL at baseline and follow-up, had a faster telomere attrition rate than the average among participants with VL <1000 RNA copies/mL. This ongoing telomere shortening is observable within a year and may lead to an increase in the risk of comorbidities and mortality later in life.

This result builds on previous investigations of TL in children with perinatally acquired HIV that identified HIV seropositivity [9, 31, 32], detectable VL [13], and being cART naive [9, 13, 32] as predictors of shorter TL. These were typically studies with participants who were younger (3.1 years [9], 13.3 years [13]), had shorter cART durations (1.2 years [9], 6.5 years [13]), and/or were cART naive. By contrast, we characterized an older cohort of children (mean, 15.5 years), who were all receiving cART and for a longer duration prior to the study enrollment (mean, 9.7 years). This likely represents the population of children and adolescents currently growing up with HIV in Africa. In the only other TL study of African CWH, the effect of HIV seropositivity (VL <400 RNA copies/mL) on shorter TL was 0.39 ln[kb/genome] [31], an unadjusted standardized effect size that is considerably greater than that in our models. However, the effect of HIV seropositivity cannot be used to infer the difference between HIV VL <60 RNA copies/mL and VL >1000 RNA copies/mL. Furthermore, our analysis benefits from sufficient power to adjust for known confounders.

Remarkably, within just 48 weeks, we measured an increased rate of telomere attrition in participants who had VL >1000 RNA copies/mL across visits compared with those who had VL <1000 RNA copies/mL across visits. There is a dearth of longitudinal telomere data in CWH; however, our data are consistent with adult studies that have demonstrated that HIV seropositivity [33] and detectable VL [34] are associated with a greater rate of attrition. The timeframes for data collection in these studies ranged from 2 years [33] to 17 years [34], compared with the 48-week period between baseline and follow-up in the VITALITY cohort. Given that telomere attrition naturally occurs at a higher rate in children than in adults [35], our longitudinal analysis therefore highlights the distinct urgency to address accelerated immune degradation in African CWH, as they may be the most vulnerable population. Achieving long-term suppression could prevent and potentially reverse accelerated TL shortening in CWH, as demonstrated in adult populations [34].

Despite cART for at least 6 months before the study, 19.5% of the VITALITY cohort had an HIV VL >60 RNA copies/mL at baseline, of which 11.6% had VL >1000 RNA copies/mL. Most participants (79%) were on a highly potent INSTI-based cART regimen, yet 4.7% of participants had VL >1000 RNA copies/mL across both visits 1 year apart, ostensibly leading to faster HIV disease progression in this subgroup. HIV disease progression has been shown to accompany an accelerated immune aging phenotype, with characteristics such as T-cell regenerative failure, genotoxicity, mitochondrial dysfunction, and chronic inflammation [5]. These changes are in turn thought to underpin the increased risk of cardiovascular [36] and renal disease [37], malignancy [38], and neurological impairments [39] in people with HIV. Therefore, this study underscores the importance of maintaining sustained viral suppression among CWH as a priority to avert or prevent progression of HIV-associated comorbidities and to promote healthy aging among CWH. Previous clinical trials have demonstrated how community-based support [40] and injectable antiretroviral therapy [41] can improve adherence and reduce virological failure in Africa. Combined with a greater emphasis on HIV drug resistance screening and cART regimen adjustments [42], these interventions could produce a dynamic multipronged approach that may achieve better outcomes in CWH.

Lower baseline CD4 T-cell count was associated with shorter TL and greater telomere attrition rate. Compared with HIV seropositivity and VL, there are fewer studies of relationships between CD4 T cells and TL, several of which have found no association [31, 32]. In some studies, proportion of CD4, rather than CD4 count, was used as the indication of disease progression, which alongside the differences in study populations may explain discrepant results. Our data imply that immune aging may be a function of disease progression as well as the current status of viral control.

Our study benefits from the inclusion of participants from 2 countries and in univariate analysis we found that Zimbabwean CWH had shorter TL than their Zambian counterparts. However, we did not adjust for the fact that Zimbabwean children were older, more often male, but less virally suppressed than Zambian children. Although evaluating differences between countries was not a primary objective, our data open the possibility that a separate biological factor might explain the relationship between HIV VL and TL that differs between study sites. There is a dearth of research into the variation of TL distribution, drivers of telomere attrition, and drug resistance mutations between countries in Africa, and this study gives the first indication that there may be heterogeneity that is relevant to how we address accelerated immune aging in these populations.

There is accumulating evidence that CMV seropositivity reduces TL among T cells [27] and is associated with an increased risk of severe non-AIDS morbidities, particularly cardiovascular and cerebrovascular events, in adults with HIV [43]. Many children in Africa are CMV seropositive within 1 year of age [12], so we investigated CMV IgG among Zimbabwean VITALITY participants as an indication of the scale of CMV infection in our cohort. We found that CMV IgG was not associated with TL or the rate of attrition. It may be the case that CMV IgG does not fully capture the effect of CMV on immune aging, so other markers, such as CMV reactivation, should be considered in future studies. Alternatively, CMV infection may not have any effect on TL in this population, but we additionally note that our statistical power was halved in this sensitivity analysis.

Given its anti-inflammatory and immunomodulatory effects, vitamin D supplementation has been proposed as an adjunct to cART [5]. This may specifically ameliorate heightened immune activation and could therefore preserve TL. A randomized controlled trial among American adults (>50 years) showed that daily vitamin D supplementation over a 5-year period significantly reduced telomere attrition at each follow-up visit within this period [19]. In our VITALITY cohort, insufficient 25(OH)D concentrations were recorded for 76.5% of participants; however, no relationship was detected between blood vitamin D concentrations and TL or its rate of attrition.

Buffy coat TL is the primary variable of interest in our study. While this enabled us to generate the largest telomere study of CWH, we are limited by a lack of investigation into complementary immune aging markers, such as epigenetic or mitochondrial markers, to support our findings. The short time period between TL measurements increases the variability of longitudinal telomere attrition rate due to short-term intraindividual TL dynamics, and we cannot compare the long-term progression of telomere attrition rate between VL groups beyond 48 weeks. Additionally, few study participants had an HIV VL 60–1000 RNA copies/mL, so the cross-sectional comparison between this group and others was less powered. It is therefore possible that differences between participants with HIV VL 60–1000 and <60 RNA copies/mL exist but were not detected in this analysis. cART regimens were not uniform among participants and specific antiretroviral agents may independently influence TL [26]. While the study was not powered to adjust for all unique cART regimens, third drug class and TDF exposure were considered in univariate analysis and no effect was detected. Our analysis is also limited by a lack of data on CMV reactivation, which could modify the relationship between TL and HIV. However, evidence for the effect of CMV on TL largely comprises studies of CMV seropositivity. It remains unknown how to best characterize the effect of CMV on immune aging in a population of African CWH with ubiquitous CMV seroprevalence. Data on other infections such as acute malaria, tuberculosis, and chronic viral hepatitis were not consistently available and thus were not considered in our analyses. These pathogens have been individually associated with TL previously [44–47], and chronic viruses may exert synergistic effects with HIV on immune aging [48].

In conclusion, our results demonstrate that HIV VL and CD4 T-cell count both correlate with TL and the rate of telomere attrition in African children growing up with HIV. Measurable immunologic deterioration within a year and the high proportion of participants with VL >60 RNA copies/mL indicate the scale of viral control challenges facing this population. Given the known long-term health implications, there is a pressing need to pay concerted attention to maintaining sustained viral suppression in CWH to ensure health longevity.

## Supplementary Material

jiag060_Supplementary_Data

## Supplementary Data

Supplementary materials are available at *The Journal of Infectious Diseases* online (http://jid.oxfordjournals.org/). Supplementary materials consist of data provided by the author that are published to benefit the reader. The posted materials are not copyedited. The contents of all supplementary data are the sole responsibility of the authors. Questions or messages regarding errors should be addressed to the author.
