# Supplementary material for: Unsuppressed Viremia and Lower CD4 Count Associated With Faster Telomere Attrition in African Children With Perinatal Human Immunodeficiency Virus on Long-term Antiretroviral Therapy
Source: J Infect Dis. 2026 Feb 7;233(6):e1407–17. doi: 10.1093/infdis/jiag060 (PMC13271384; doi:10.1093/infdis/jiag060)
Supplement: jiag060_Supplementary_Data [file jiag060_supplementary_data.docx]

**Supplemental data**

**Table S1.** Univariate analysis of the association between potential explanatory variables and TL*.*

|  | **β** | **P-value** |
| --- | --- | --- |
| **Age** | -0.032 (-0.058, -0.006) | 0.016^a^ |
| **Sex [ref females]** | -0.247 (-0.381, 0.112) | <0.001^b^ |
| **Body mass index** | -0.003 (-0.027, 0.0215) | 0.825^a^ |
| **Age at cART initiation** | 0.013 (-0.003, 0.029) | 0.116^a^ |
| **cART regimen (third drug class)** |  | 0.409^c^ |
| **PI** | -0.085 (-0.279, 0.109) |  |
| **NRTI/NNRTI** | 0.119 (-0.090, 0.328) |  |
| **INSTI** | -1.13 x10^-4^ (-0.077, 0.077) |  |
| **TDF in regimen [ref non-TDF]** | -0.028 (-0.205, 0.119) | 0.600^b^ |
| **SES** |  | 0.693^c^ |
| **I** | -0.061 (-0.220, 0.099) |  |
| **II** | -0.012 (-0.168, 0.144) |  |
| **III** | 0.125 (-0.012, 0.262) |  |
| **IV** | -0.078 (-0.231, 0.074) |  |
| **V** | 0.019 (-0.131, 0.168) |  |
| **25(OH)D [ref ≥75nmol/L]** | -0.017 (-0.176, 0.142) | 0.833^b^ |
| **Study site [ref Zambia]** | -0.218 (-0.353, -0.084) | 0.002^b^ |

*Slope or β and 95% confidence interval (CI) shown alongside p-value. A negative β represents*

*association with a shorter TL. P-values: ^a^Pearson’s correlation, ^b^unpaired t-test, ^c^ANOVA.*

*Abbreviations: cART – combination antiretroviral therapy, INSTI – integrase strand transfer inhibitor, NRTI – nucleoside reverse transcriptase inhibitor, NNRTI – non-nucleoside reverse transcriptase inhibitor, PI – protease inhibitor, SES – socioeconomic status, TDF – tenofovir disoproxil fumarate, TL – telomere length, 25(OH)D_3_ – blood 25-hydroxy vitamin D levels.*

**Table S2**. Study participant characteristics for the VITALITY participants with longitudinal data (N=783).

|  | **S 🡪 S** (N=648, 82.8%) | **S 🡪 U** (N=50, 6.4%) | **U 🡪 S** (N=48, 6.1%) | **U 🡪 U** (N=37, 4.7%) | **P-value^b^** |
| --- | --- | --- | --- | --- | --- |
|  |  |  |  |  |  |
| **Age, years** | 15 ±3 (11,20) | 15 ±2 (11,19) | 16 ±3 (11,20) | 17 ±2 (11,20) | 0.004^a^ |
| **Female sex** | 363 (56) | 22 (44) | 19 (40) | 15 (41) | 0.021^b^ |
| **Body mass index, kg/m^2^** | 18 ±3 (13,30) (N=646) | 18 ±3 (12,30) | 18 ±3 (13,24) | 19 ±3 (13,27) | 0.992^a^ |
| **HIV VL RNA copies/mL** | N/A | N/A | 15571 [4812,42020] (1015,487652) | 13900 [5030,43610] (1228,7848680) (N=37) | 0.929^c^ |
| **Current CD4 count/µl blood** | 617 ±230 (38,1631) (N=647) | 564 ±248 (7,1049) (N=48) | 413 ±222 (27,948) (N=47) | 332 ±182 (27,821) | <0.001^a^ |
| **Age at cART initiation, years** | 5 [2, 9] (0,19) | 4 [2,8] (0, 15) | 6 [3, 8] (0, 15) | 6 [4, 10] (0,15) | 0.146^d^ |
| **Duration of cART, years** | 10 ±4 (1,19) | 10 ±4 (2, 17) | 10 ±3 (2, 16) | 10 ±4 (2, 17) | 0.646^d^ |
| **cART regimen** |  |  |  |  | <0.001^b^ |
| **PI** | 59 (9) | 7 (14) | 16 (33) | 11 (30) |  |
| **NRTI/NNRTI** | 47 (7) | 4 (8) | 9 (19) | 8 (22) |  |
| **INSTI** | 542 (84) | 39 (78) | 23 (48) | 18 (49) |  |
| **TDF** |  |  |  |  | <0.001^b^ |
| **TDF in regimen** | 551 (85) | 37 (74) | 29 (60) | 20 (54) |  |
| **CMV IgG, IU/mL** | 31 [27, 36] (18, 51) (N=322) | 31 [26, 33] (19, 45) (N=23) | 32 [29, 36] (23, 48) (N=32) | 34 [29, 43] (22, 48) (N=19) | 0.094^d^ |
| **SES** |  |  |  |  | 0.058^b^ |
| **I** | 127 (20) | 9 (18) | 16 (33) | 6 (16) |  |
| **II** | 121 (19) | 11 (22) | 10 (21) | 11 (30) |  |
| **III** | 148 (23) | 8 (16) | 4 (8) | 7 (19) |  |
| **IV** | 137 (21) | 7 (14) | 7 (15) | 4 (11) |  |
| **V** | 115 (18) | 15 (30) | 11 (23) | 9 (24) |  |
| **Vitamin D, nmol/L 25(OH)D3** | 64 [54, 74] (21, 139) | 61 [53, 71] (29, 145) | 71 [57, 91] (27, 135) | 64 [55, 67] (37, 91) | 0.106^d^ |
| **≥75** | 184 (24) | 8 (16) | 20 (42) | 6 (16) | 0.011^b^ |
| **<75** | 494 (76) | 42 (84) | 28 (58) | 31 (84) |  |
| **Zambian study site** | 326 (50) | 27 (54) | 16 (33) | 18 (49) | 0.133^b^ |

*Data were either presented as N (%), median [IQR](range), or mean ±SD(range). P-values: ^a^ANOVA,* ^b^*Chi squared, ^c^Mann-Whitney, ^d^Kruskal-Wallis. Abbreviations: cART – combination antiretroviral therapy, CD4 count – blood CD4^+^ T-cell count, CMV – cytomegalovirus, INSTI – integrase strand transfer inhibitor, NRTI – nucleoside reverse transcriptase inhibitor, NNRTI – non-nucleoside reverse transcriptase inhibitor, PI – protease inhibitor, SES – socioeconomic status, S – suppressed, U – unsuppressed, TDF – tenofovir disoproxil fumarate, VL – viral load, 25(OH)D_3_ – blood 25-hydroxy vitamin D levels.*

**Table S3.** Univariate analysis of the association between potential explanatory variables and telomere attrition rate.

|  | **β** | **P-value** |
| --- | --- | --- |
| **Age** | -0.003 (-0.030, 0.024) | 0.831^a^ |
| **Sex [ref females]** | 0.002 (-0.139, 0.143) | 0.976^b^ |
| **Body mass index** | -0.010 (-0.036, 0.015) | 0.432^a^ |
| **Age at cART initiation** | -0.004 (-0.096, 0.142) | 0.637^a^ |
| **cART regimen (third drug class)** |  | 0.169^c^ |
| **PI** | -0.051 (-0.250, 0.149) |  |
| **NRTI/NNRTI** | -0.202 (-0.425, 0.021) |  |
| **INSTI** | 0.030 (-0.050, 0.109) |  |
| **TDF in regimen [ref non-TDF]** | 0.029 (-0.145, 0.203) | 0.743^b^ |
| **SES** |  | 0.785^c^ |
| **I** | 0.049 (-0.110, 0.208) |  |
| **II** | -0.043 (-0.199, 0.112) |  |
| **III** | -0.021 (-0.158, 0.117) |  |
| **IV** | 0.041 (-0.124, 0.206) |  |
| **V** | -0.027 (-0.196, 0.141) |  |
| **25(OH)D [ref ≥75nmol/L]** | 0.061 (-0.100, 0.222) | 0.455^b^ |
| **Study site [ref Zambia]** | 0.345 (0.207, 0.483) | <0.001^b^ |
| **Baseline TL** | -0.576 (-0.634, -0.519) | <0.001^a^ |

*β and 95% confidence interval (CI) shown alongside p-value. A negative β represents association with a shorter TL. P-values: ^a^Pearson’s correlation, ^b^unpaired t-test, ^c^ANOVA. Abbreviations: cART – combination antiretroviral therapy, INSTI – integrase strand transfer inhibitor, NRTI – nucleoside reverse transcriptase inhibitor, NNRTI – non-nucleoside reverse transcriptase inhibitor, PI – protease inhibitor, SES – socioeconomic status, TDF – tenofovir disoproxil fumarate, TL – telomere length, 25(OH)D_3_ – blood 25-hydroxy vitamin D levels.*

**Table S4.** Multivariable linear regression sensitivity analysis of telomere attrition rate adjusted for regression to the mean with HIV VL and CD4 T cell count.

|  | **HIV VL model** | | | | **CD4 count model** | | | |
| --- | --- | --- | --- | --- | --- | --- | --- | --- |
|  | **Unadjusted** | | **Adjusted**^a^ | | **Unadjusted** | | **Adjusted**^a^ | |
|  | **β** | **P-value** | **β** | **P-value** | **β** | **P-value** | **β** | **P-value** |
| **HIV viral load trajectory**  **[ref S 🡪 S]** |  |  |  |  |  |  |  |  |
| **S 🡪 U** | -0.025  (-0.312, 0.263) | 0.867 | -0.001  (-0.287, 0.284) | 0.993 |  |  |  |  |
| **U 🡪 S** | -0.007  (-0.301, 0.286) | 0.961 | -0.008  (-0.301, 0.284) | 0.956 |  |  |  |  |
| **U 🡪 U** | -0.387  (-0.719, -0.056) | 0.022 | -0.336  (-0.666, -0.005) | 0.047 |  |  |  |  |
| **CD4 count**  **(per 100 cells/μL)** |  |  |  |  | -0.021  (-0.051, 0.008) | 0.624 | 0.040  (0.010, 0.070) | 0.009 |

^a^*Adjusting for age, sex, baseline TL and site. β and 95% confidence interval (CI) shown alongside p-value. A negative β represents association with a greater rate of telomere attrition. Abbreviations: S – suppressed, U – unsuppressed, TL – telomere length, VL – viral load.*
